# Outcome in young adults who were diagnosed with complex regional pain syndrome in childhood and adolescence

**DOI:** 10.1097/PR9.0000000000000860

**Published:** 2020-10-12

**Authors:** Becky J. Wong, Isabel A. Yoon, Elliot J. Krane

**Affiliations:** a Stanford University School of Medicine, Stanford, CA, USA; b Stanford Children's Health and the Packard Children's Hospital at Stanford, Stanford, CA, USA

**Keywords:** Pain, Neuropathic, CRPS, Child, Pediatric

## Abstract

**Introduction:**

Complex regional pain syndrome (CRPS) is a neuropathic pain condition of unknown etiology. Little is known of long-term outcomes of young adults who were diagnosed with CRPS as children.

**Methods:**

In this study, surveys were mailed to adults who were treated for childhood CRPS at the Lucile Packard Children's Hospital between 1994 and 2018. Completed surveys were analyzed for pain symptoms. Health-related quality-of-life surveys, the Optum SF-8, were analyzed based on norm-based scoring.

**Results:**

This study had a 50% response rate. Patients were treated with physical and occupational therapy, peripheral or sympathetic nerve blocks, medication for neuropathic pain, and psychotherapy. Sixty-eight percent of the subjects reported pain. Each 1-year increase in the patient's age at the time of CRPS diagnosis increased the odds of having at least mild pain as an adult by 61% (*P* = 0.005). Most patients had slightly lower quality-of-life scores than the US population average in both the mental component score (43.4, 95%, confidence interval 3.4) and the physical component score (44.4, 95%, confidence interval 3.3).

**Conclusions:**

Young adults in our sample had long-lasting pain symptoms. More than two-thirds of adult patients reported some degree of pain, and these patients had a lower quality of life. Encouraging was that the majority did not have CRPS spreading to other areas, and their pain did not warrant further treatment. Understanding long-term outcomes may lead to risk stratification earlier in the disease to improve future quality of life.

## Abstract 2

Supplemental Digital Content is Available in the Text.

Most patients with childhood-onset complex regional pain syndrome continue to have some complex regional pain syndrome–related pain into young adulthood and have a lower health-related quality of life.

## 1. Introduction

Complex regional pain syndrome (CRPS) is a spontaneous or evoked disease state which results in longstanding, severe, spontaneous physical pain, hyperalgesia and allodynia, sensory distortion, and perfusion alteration of the affected body part, which is generally 1 or more limbs. Pediatric cases of CRPS typically begin in adolescence with a predominance in females,^17^ which may be related to direct or epigenetic hormonal effects that are associated with puberty. The biological basis of CRPS is poorly understood, and CRPS remains a clinical diagnosis. Symptoms of CRPS typically persist long after any inciting injury has healed, and there remains vigorous debate regarding the best treatment of CRPS in both children^4^ and adults.^6^

There has been substantial clinical and basic research on CRPS, but very little focus on childhood CRPS, which behaves differently than CRPS in adults. Although the short-term prognosis of pediatric CRPS is believed to be better than that of adult CRPS, with many, perhaps most children returning to a state of no or little pain and full function, less is known of the long-term outcome of pediatric CRPS in adulthood. Some sources have cited resolution of symptoms in children with CRPS to be as high as 50% to 90%.^3^ However, a retrospective follow-up study of patients younger than 16 years showed a less favorable disease course comparable with that in adults.^20^

The primary objective of this study was to identify and characterize recurrent or persistent symptoms of pain or physical disability in adults who were diagnosed with CRPS and treated during childhood or adolescence. The secondary objective was to correlate the presence of pain as adults with age at diagnosis, sex, race, and time elapsed since treatment of childhood CRPS. We hypothesized that there would be a lesser incidence of chronic pain in the study population compared with adult-onset CRPS.

This information is potentially important in predicting long-term morbidity and complications from this disease to allow pediatric patients and their parents to have accurate expectations when given their diagnosis. Better understanding of long-term outcomes will also assist clinicians to subgroup children with CRPS into prognostic categories, and presumably lead to more specific treatment paradigms and follow-up, such as focusing on implementing long-term physical therapy and/or psychological counseling earlier in the course of the disease.

## 2. Methods

This study was approved by the institutional review board. We defined pediatric CRPS as a diagnosis of CRPS made in our pediatric pain management clinic by faculty pediatric pain specialists using established contemporaneous international criteria for the diagnosis of CRPS^12^ in patients who were younger than 18 years at the onset of their symptoms and at the time of the initiation of their treatment. Potential study subjects were identified through a medical record database search of all patients treated in the pain management clinic between the years 1994 and 2018 and whose charts were *ICD* coded with the diagnosis of CRPS type 1 or type 2. Chart notes were reviewed by one of the investigators to confirm the veracity of the coded diagnosis of CRPS.

Potential subjects who were older than 18 years at the time of identification were asked to agree to participation in this study using a cover letter and survey sent by the United States Postal Service to their last known address. Those who did not reply to the mailed request within 4 weeks were contacted by e-mail, if possible, and/or by telephone. Participation was voluntary subsequent to completion of written informed consent; no financial incentives for participation in the study were offered or provided.

Data were collected using a survey of specific questions. Subjects completed either a paper or electronic questionnaire, or in the case of some participants contacted by telephone, the survey was administered by telephone if they so chose, with responses recorded by a research associate. Data were then entered into a secure encrypted online database (REDCap). For those potential subjects identified who did not respond to mailed or e-mailed requests, and who could not be reached telephonically, we performed a national search of death certificate records and published obituaries using Google Search to determine whether these subjects were known to have died.

Patients were mailed a 2-part survey (see Appendix, http://links.lww.com/PR9/A83). The first part of the questionnaire included a validated survey of health-related quality of life, the Short Form-8 (SF-8) Health Survey. The second part of the survey consisted of general questions focused on the current state of the previously affected CRPS limb, if the symptoms had spread and if the patients had sought additional therapies beyond their initial treatment of CRPS at first diagnosis. The presence of pain was queried in both the SF-8 survey and in the second part of the survey. In the SF-8 survey, the question related to general bodily pain and was used for the composite physical score. The pain question in the second part of the survey was specific to the CRPS-affected limb and was used for analysis of pain vs no pain (see Appendix, http://links.lww.com/PR9/A83).

### 2.1. SF-8 Health Survey

There are several forms of the SF Health Surveys, including forms based on 8-, 10-, 12-, and 36-question surveys. Specifically, the SF-8 is a widely used survey^23^ composed of 8 questions designed to quantify a health-related quality-of-life score. The SF survey has been applied to a wide range of patient populations including patients with migraine,^21^ patients with chronic heart failure,^10^ older patients with psychiatric diseases,^19^ and patients with diabetes.^7^

To create the norm-based scoring, Optum surveyed a random sample of 7,472 individuals from the general US population with the SF-8 questionnaire and calculated normative scores based on those survey mean values and SDs.^23^ Thus, using scoring software purchased through Optum, we report our study population results in summary scales of the physical component score (PCS) and the mental component score (MCS) which have been normalized to the US population. The scores are scaled based on norm-based scoring (NBS). In NBS, each scale is scaled to have a mean of 50 and an SD of 10, in which each point equals one-tenth of an SD. When a participant's scale score is less than 45 or a group mean scale score is below 47, health status is below the average range.^1,16^ The general population norm is built into the scoring algorithm for NBS and simplifies the interpretation.

The Optum SF-8 Health Survey measures 8 health domains and is scored using norm-based methods. The PCS is more heavily composed of categories such as physical functioning, physical role, bodily pain, and general health, while the MCS is more heavily composed of vitality, social functioning, emotional role, and mental health.^22^

Because this is a descriptive-based study, prestudy power analysis was not performed. Parametric and nonparametric variables were compared using single-tailed Student *t* tests with Bonferroni corrections for multiple comparisons. Statistical significance was defined as *P* < 0.05. Univariate analysis was performed based on the Fisher exact test for predictors of sex, race (white and nonwhite), and ethnicity and based on the Wilcoxon test for the predictors of age at CRPS diagnosis and time elapsed from diagnosis to survey.

## 3. Results

**Figure 1.**
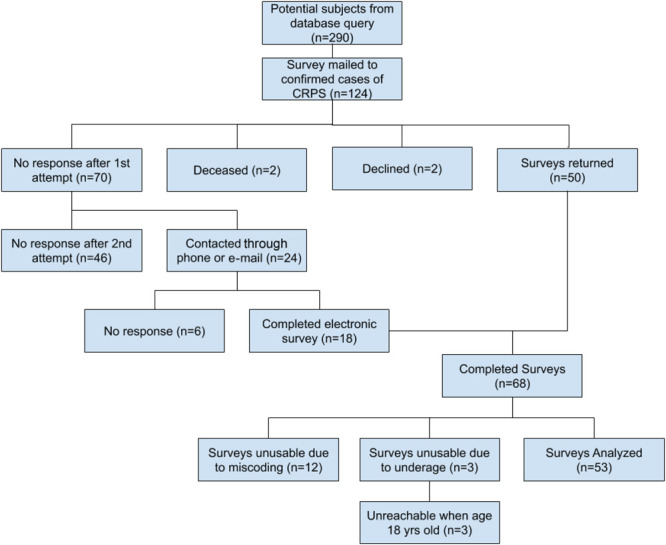
Study subjects identified, contacted, consenting, and responding with completed surveys.

A medical record database query of patients coded for CRPS was performed. Patients identified to represent cases of CRPS, were mailed requests for participation, informed consent forms, and the survey by United States Postal Service mail. A post hoc audit of the 124 surveys found that 12 subjects had diagnoses which had been miscoded and 3 subjects were younger than 18 years at the time of mailing. These surveys were disqualified from analysis. Two former patients were subsequently identified as deceased. In total, 53 surveys were analyzed of 107 valid surveys distributed (Fig. 1), for a response rate of 50%. In survey completers, the mean follow-up time was 6.0 years, with a range of 19 months to 13.5 years (Table 1).

**Table 1:** Patient demographics.

Age	Range	Mean ± SD
Age at diagnosis (yr)	9–17	13.5 ± 2.2
Age at survey (yr)	18–27	19.1 ± 2.0
Time from treatment to survey (yr)	1.6–13.5	6.0 ± 3.0
	**Male (%)**	**Female (%)**
Sex	12 (23%)	41 (77%)
Race		
White	8 (66.7%)	32 (78.0%)
Asian	2 (16.7%)	2 (4.9%)
African American	0	2 (4.9%)
Native American	0	1 (2.4%)
Other	2 (16.7%)	2 (4.9%)
Unknown	0	2 (4.9%)
Ethnicity		
Non-Hispanic	9 (75.0%)	32 (78.0%)
Hispanic or Latino	3 (25.0%)	4 (9.8%)
Unknown	0	5 (12.2%)

All 53 study patients received a multidisciplinary model of treatment as is the standard of care for pediatric CRPS consisting of frequent and rigorous physical therapy, occupational therapy (OT) including limb desensitization, psychotherapy, and supplementation with interventional and pharmacologic therapies. Thirty-two patients (60%) were treated only as outpatients and 21 patients (40%) initially as outpatients and subsequently as inpatients before returning to outpatient management. Forty patients (76%) received peripheral and/or sympathetic nerve blocks, all 53 patients received PT, and 44 patients (83%) received psychotherapy. Forty-one patients (77%) were prescribed gabapentin and 43 (81%) tricyclic or serotonin–norepinephrine reuptake inhibitor antidepressants. Opioids were not prescribed to patients unless they were taking opioids at the time of referral to the practice, in which case they were tapered and discontinued as rapidly as tolerated.

**Figure 2.**
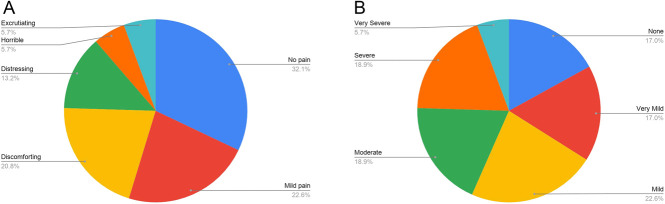
(A) Pain level in the CRPS limb. (B) General pain level in body. CRPS, complex regional pain syndrome.

Of the 53 study patients, 68% reported some degree of pain at the time of the survey, with only 32% having spread of CRPS symptoms to other body parts, and 38% having recurrent CRPS symptoms requiring treatment. Figure 2A and B gives the breakdown of pain levels in the CRPS limb and general body pain, respectively. There was no significant association between the incidence of pain and race (white vs other), ethnicity (Hispanic vs non-Hispanic), or the interval duration from treatment to survey completion (the Fisher exact test for predictors of sex, white, and Hispanic race; the Wilcoxon test for predictors of age at CRPS diagnosis and of time from diagnosis to survey). Notably, there was an absence of African American in the study sample. In general, the population of African Americans in our clinic referral community was small. Only age at CRPS diagnosis vs pain in early adulthood at the time of survey had a statistically significant association (*P* < 0.05, the Wilcoxon ranked-sum test) with or without adjustment for sex, race, or ethnicity (Table 2).

**Table 2:** Pain vs No Pain in Early Adulthood.

Characteristic	Pain	No pain
No. of patients	36	17
Sex (female)	73.2%	26.8%
Sex (male)	50.0%	50.0%
Race (white)	70.0%	30.0%
Race (nonwhite)	61.5%	38.5%
Ethnicity (Hispanic)	66.7%	33.3%
Ethnicity (non-Hispanic)	68.3%	31.7%
Median age at CRPS diagnosis (y)	14.0*	12.0*
Median time from diagnosis to survey (y)	4.4	7.2
PCS (SD)	39.3 (10.8)	55.7 (4.0)
MCS (SD)	40.9 (11.7)	48.83 (12.6)

* *P* < 0.05, the Wilcoxon ranked-sum test.

CRPS, complex regional pain syndrome; MCS, mental component score; PCS, physical component score.

Using logistic regression for age at CRPS diagnosis, we found that each increment of 1 year of age at diagnosis increased the odds of having some pain at the time of the survey by a factor of 1.61, an increase of 61% (*P* = 0.005) per year of age. Neither age at the time of CRPS diagnosis nor the amount of time that had elapsed between diagnosis and the survey was a significant predictor of how much pain or what level of pain would be reported.

The SF-8–scaled composite scores for health-related quality of life were 44.4 (95% confidence interval [CI] 3.3) for the PCS and 43.4 (95% CI 3.4) for the MCS. Although not statistically significant, there was a trend suggesting that young adult males fared better than females who had had childhood CRPS.

As a subset analysis, we analyzed patients who were aged 21 years or older at their survey completion. There were 10 subjects with an average of 9.3 years since their CRPS diagnosis. There was a small majority of patients who reported some pain in their CRPS limb, and most patients had at least some bodily pain (Table 3). We also analyzed the 29 study patients for whom it had been at least 5 years since their CRPS diagnosis. Although this was a small sample size, it showed that with increasing age at diagnosis, the pain score at the time of the survey also increased (Supplemental Figure 1, available at http://links.lww.com/PR9/A83).

**Table 3:** Subset analysis of pain in specific groups.

	All patients	Age ≥21 y at survey	Time ≥5 y since diagnosis	Patients on opioids during treatment
No. of patients	53	10	29	26
Mean age at diagnosis (yrs)	13.5	13.3	12	14.2
Mean y since diagnosis	6.0	9.3	8.2	5.3
Pain in the CRPS limb				
None	32.1%	40%	44.8%	15.4%
Mild	22.6%	50%	17.2%	19.2%
Discomforting	20.8%	10%	13.8%	30.8%
Distressing	13.2%	0	6.9%	23.1%
Horrible	5.7%	0	6.9%	3.8%
Excruciating	5.7%	0	10.3%	7.7%
Bodily pain				
None	17.0%	10.0%	24.1%	11.5%
Very mild	17.0%	40.0%	13.8%	11.5%
Mild	22.6%	30.0%	24.1%	7.7%
Moderate	18.9%	10.0%	10.3%	30.8%
Severe	18.9%	0	13.8%	26.9%
Very severe	5.7%	10.0%	13.8%	11.5%
PCS (mean, SD)	44.4 (12.0)	50.26 (10.7)	45.41 (13.0)	40.17 (11.3)
MCS (mean, SD)	43.4 (12.4)	50.17 (10.3)	44.75 (11.9)	39.13 (13.8)

CRPS, complex regional pain syndrome; MCS, mental component score; PCS, physical component score.

Twenty-six patients were on 1 or more of the following opioids at the time of the survey: hydrocodone, hydrocodone/acetaminophen, tramadol, oxycodone, methadone, morphine, and fentanyl. Although this subset of patients is small, we see general trends that these patients who were on opioids reported more pain in the CRPS limb as well as more severe overall body pain (Table 3).

Regarding physical functioning, Supplemental Figure 2a (available at http://links.lww.com/PR9/A83) shows to what degree the patients' physical activities and function were limited by their CRPS condition. The scores for the SF-8 survey question #2 (physical activities such as walking and climbing stairs) and SF-8 survey question #3 (function in daily work at home and away from home) were averaged. The patients were separated by whether or not they had reported any pain or no pain in their CRPS limb. A similar analysis is seen in Supplemental Figure 2b, regarding how affected patients were by depression or anxiety and how these may have impaired function at school or work (available at http://links.lww.com/PR9/A83). This was an average of data from SF-8 survey questions #6, #7, and #8 which asked how emotional problems limited social activities with family and friends, how much patients had been bothered by feelings of anxiety or depression, and how emotional problems have inhibited work or school activities. Again, the patients were sorted by whether or not they reported current pain in the CRPS limb. Finally, we reviewed the patients' medical files to record the health and presence or absence of CRPS symptoms at the time of their last pain clinic visit (Supplemental Table 1, available at http://links.lww.com/PR9/A83).

## 4. Discussion

We surveyed adult patients who had the diagnosis of CRPS during childhood or adolescence treated in our institution from 1994 until 2018. We found that contrary to the common impression that the pediatric patients with CRPS usually recover without residual pain or disability, the majority (68%) of patients completing our survey reported CRPS-related pain at levels classified from “mild” to “excruciating.” Furthermore, in our subset of study patients, we found that the younger a child was at the age of diagnosis of CRPS, the less their likelihood of having pain later in life; each increasing year of age at the time of diagnosis increased the odds of a report of pain at the time of this survey by about 60%. Yet, we were pleased to observe that a minority of patients (32%) in our sample had symptoms and signs of CRPS that had spread to other limbs, and only 38% of patients responding to our questionnaire felt that their symptoms warranted further evaluation and treatment at the time of our survey.

The long-term outcome of CRPS in adults has been more extensively studied^8^ than pediatric CRPS patient outcomes. In 1 systematic review of adult CRPS type 1 patients, Bean et al. found that in 3 prospective studies, symptoms improve markedly within 6 to 13 months of onset for many patients. However, in 12 retrospective studies with highly heterogeneous findings, there were lasting impairments in patients.^2^ The pediatric CRPS literature has shown relapse rates of 29% to 37%,^5,13,18^ and high rates of full recovery with 1 study by Sherry et al. showing 88% complete resolution in pediatric patients^18^ after intense inpatient PT (5–6 hours daily for up to 14 days), with reinstitution of the exercise program if there was a relapse. Brooke et al. performed a study which similarly reported an 87% resolution rate^5^ with inpatient treatment but with a shorter follow-up time averaging only 21 months. Sherry et al. and Brooke et al. did not use nerve blocks as part of their treatments. These studies differed from our study in the therapies used to treat CRPS. In the other studies, patients were treated daily in an intensive program of PT, OT, and, in some patients, psychological therapy. It has been postulated that these modalities may allow patients to be more of a driver in their treatments and may be a reason for observed differences to our study. In our study, our patients had a more multimodal approach with medication and nerve blocks when indicated to support aggressive PT, OT, as well as psychological therapy. Furthermore, we report here longer periods of follow-up than either the Sherry or Brooke studies.

In our study group, pediatric patients diagnosed with CRPS at an older age were more likely to have pain as adults. The effect of age at onset of CRPS on the likelihood of pain in adulthood has been explained by the perception that younger patients demonstrate greater willingness to participate in appropriately targeted treatments,^24^ but this is in variance with our consistent observation that convincing younger children to participate in painful PT is very difficult compared with teens, leading us to hypothesize that the relationship of age at onset and pain in later life represents a biological phenomenon rather than a behavioral one.

Although most study patients with childhood CRPS have pain as an adult, there is a less clear predictive value of the amount of pain each patient will experience as an adult. Regardless of the degree of pain suffered in later years, disease persistence, progression, and pain are multifactorial. Ongoing family support, the patient's psychological substrate, genetic vulnerability, and unique biology as well as additional treatments as adults and general learned coping skills all play a role in the degree of pain a patient reports in later years of life.

The SF-8 Health Survey by Optum is a survey that is used to assess overall quality of life in chronically ill populations.^15,23^ The SF-8 survey has been used and validated for patients with a wide variety of medical conditions including migraine,^21^ chronic heart failure,^10^ psychiatric diseases,^19^ and diabetes,^7^ and here in this study, we have used it for our patients who have had childhood CRPS. When Optum created the SF-8 scoring tool, it surveyed a sample of the US population which also likely had a myriad of different diseases and health conditions. Therefore, the use of a standard measure such as the SF-8 allows reporting on the status of patients with CRPS using a standardized and validated measure. Our subjects had lower SF-8 scores in both the composite PCS of 44.4 (95%, CI 3.3) and the composite MCS of 43.4 (95%, CI 3.4), which are below average. These quality-of-life scores are congruent with our finding of pain in the subjects' young adult years. Similar results have been found in a wide variety of childhood diseases that have lasting symptoms that also decrease the quality of life in adulthood.^9,11,14^ We believe that the residual pain in our cohort may lead to mental fatigue, emotional distress, and social isolation from curtailment of activities or employment, which all contribute to lower quality-of-life scores. Although these data are telling of young adults who were diagnosed with childhood CRPS, we are unable to predict the long-term outcomes in 1 or 2 more decades. This would be the subject of future studies to survey patients longitudinally for many more years.

In the small subset of 26 patients on opioids, there was a trend to report more pain and the reason is likely multifactorial: Patients with more severe disease may be more likely to be prescribed opioids. Opioids are not the best treatment for CRPS, thus perpetuating the cycle of pain and opioid use. We were able to note functional status by examining certain SF-8 survey questions. In general and as expected, patients with pain were more restricted in their physical function compared with patients who did not report pain. From a social and emotional standpoint, we looked at SF-8 survey questions related to patients' social activities, personal problems, and feelings of anxiety affecting their lives. Again, our data showed that patients who reported pain in their CRPS limb were generally more emotionally affected at the time of survey. To keep the surveys to a manageable number of questions, we did not directly query patients on other pain states or nonpain psychopathology such as suicidality, eating disorders, headaches, or abdominal pain. These comorbid conditions are relevant to the overall quality of life for these CRPS survivors and warrant future study.

We reviewed the medical record notes of the last clinic visit for each of the patients to detect a correlation between the amount of pain when last seen in clinic and later pain outcome. Many patients were seen to have symptoms at their last visit, yet did not subsequently return to the clinic. Reasons for this are many, including feeling well so they did not seek any follow-up, feeling too poorly to tolerate another clinic appointment or so that they sought an alternative provider, return to their hometown clinic, departure to attend college, or due to family move. There is, as expected, a general trend of having a lower health-related quality-of-life score (by the MCS and PCS) if the patient had pain or comorbid conditions at their last pain clinic appointment. However, with such a small sample size for each category of pain at their last clinic appointment, further studies are needed to truly understand the correlation.

### 4.1. Limitations

**Figure 3.**
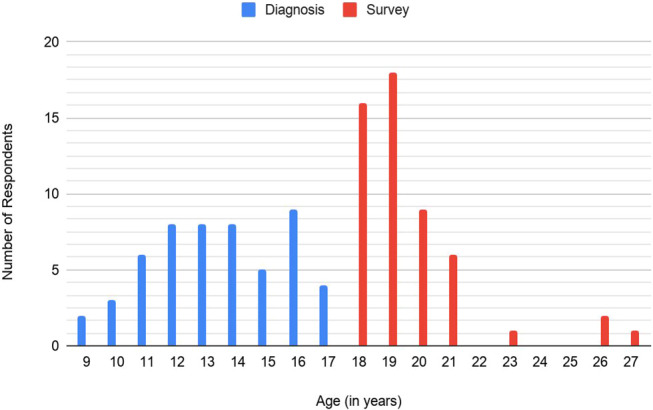
Distribution of ages at CRPS diagnosis and at survey completion. CRPS, complex regional pain syndrome.

Data quality of all surveys is limited by the response rate and self-selection bias. It is possible that patients with ongoing symptoms related to CRPS may be more, or less, likely to participate in the survey than patients who do not have ongoing pain, which would lead to overreporting or underreporting of the incidence of pain persistence. Our series also had a preponderance of survey respondents in early adulthood ages of 18 to 21 years vs patients in the third or fourth decade of life, despite cases of childhood CRPS from as early as 1994 (Fig. 3). There was a mean follow-up time of 6 years with the longest being 13.5 years. We attributed this to an absence of accurate contact information (physical address, e-mail address, and phone number) to which we could send surveys or contact subjects by telephone. Thus, the older the patient, the more likely that the patient will have relocated, making contact impossible. It therefore is possible that we have underreported or overreported persistent or recurrent symptoms of CRPS over time. Furthermore, most study participants were Anglo-American, which may reflect a higher incidence of CRPS in this racial group, or the demographic characteristic of our clinic population.

Of the 46 potential subjects who did not respond to mailings and who could not be contacted by telephone or electronic mail, a mean of 5 years were found to have passed in the interval between their CRPS treatment and the time of this study, and that they were similar in CRPS diagnosis and treatment, age, ethnicity, and sex compared with the survey responders. Therefore, we do not believe that there were any systematic differences between the responder and nonresponder cohorts.

Although this study used the presence of pain and the SF-8–scaled scores for health-related quality of life, we did not include survey questions to elucidate other manifestations of nonpain pathological outcomes such as abdominal pain, neurological disorders, suicidality, or eating disorders. As with any survey, there is room for misinterpretation of the question by the survey taker. For example, the question of CRPS-related pain on page 4 of the survey (see Appendix, http://links.lww.com/PR9/A83) might have been misunderstood to be asking about any pain in the body, not just pain in the CRPS limb. Unfortunately, it is impossible to know whether a subject interpreted the question in the way that it was originally intended.

## 5. Conclusions

Complex regional pain syndrome is a profoundly painful condition. In children, the short-term prognosis of CRPS for reduction or elimination of pain and restoration of function is considered by pediatric pain specialists to be very good in nearly all cases. Here, we demonstrate by a survey of a sample of young adults who were treated for CRPS in their childhood or adolescent years that, in fact, a minority of former pediatric CRPS patients are completely symptom- or disease-free in their adulthood, and that these young adults who had childhood CRPS have a lower quality-of-life score compared with a general US population sample. As there is a dearth of long-term outcomes data on pediatric CRPS, we believe that this small study should prompt larger prospective, longitudinal studies of adult survivors of childhood CRPS to include analysis of functional abilities, comorbid disease states, and recurrent or persistent CRPS pain. Awareness of these findings is important for patients and their parents, so that they understand the expected trajectory of CRPS and to inspire more investigations to develop better treatment paradigms.

## Disclosures

The authors have no conflicts of interest to declare.

## Appendix A. Supplemental digital content

Supplemental digital content associated with this article can be found online at http://links.lww.com/PR9/A83.

## Supplementary Material

SUPPLEMENTARY MATERIAL

## References

[1] Advantages of norm based scoring. In: Optum^®^ user's manual for the SF-36v2 health survey. 2nd ed Chapter 7. p. 81–4. Available at: https://cdn-aem.optum.com/content/dam/optum/resources/Manual%20Excerpts/Norm-based%20Scoring%20(NBS).pdf. Accessed October 15, 2019.

[2] Bean DJ Johnson MH Kydd RR The outcome of complex regional pain syndrome type 1: a systematic review. J Pain 2014;159:677–90. 10.1016/j.jpain.2014.01.500 24530407

[3] Binder A Schattschneider J Baron R Complex regional pain syndrome type I (reflex sympathetic dystrophy). In: Waldman SD, editor. Pain management. 2nd ed Philadelphia: Saunders, 2011 p. 272–89.

[4] Borucki AN Greco CD An update on complex regional pain syndromes in children and adolescents. Curr Opin Pediatr 2015;27:448–52. 26087424 10.1097/MOP.0000000000000250

[5] Brooke V Janselewitz S Outcomes of children with complex regional pain syndrome after intensive inpatient rehabilitation. PMR 2012;4:349–54. 10.1016/j.pmrj.2012.01.014 22465690

[6] Bruehl S Complex regional pain syndrome. BMJ 2015;351:H2730. 26224572 10.1136/bmj.h2730

[7] Camacho F Anderson RT Bell RA Goff DC Jr Duren-Winfield V Doss DD Balkrishnan R Investigating correlates of health-related quality of life in a low-income sample of patients with diabetes. Qual Life Res 2002;11:783–96. 12482162 10.1023/a:1020858102483

[8] de Mos M Huygen FJ van der Hoeven-Borgman M Dieleman JP Ch Stricker BH Sturkenboom MC Outcome of the complex regional pain syndrome. Clin J Pain 2009;25:590–7. 19692800 10.1097/AJP.0b013e3181a11623

[9] Ehrhardt MJ Mulrooney DA Baassiri MJ Bjornard K Sandlund JT Brinkman TM Neurocognitive, psychosocial, and quality-of-life outcomes in adult survivors of childhood non-Hodgkin lymphoma. Cancer 2018;124:417–25. 28915338 10.1002/cncr.31019 PMC5760296

[10] Ekman I Fagerberg B Lundman B Health-related quality of life and sense of coherence among elderly patients with severe chronic heart failure in comparison with healthy controls. Heart Lung 2002;31:94–101. 11910384 10.1067/mhl.2002.122821

[11] Gunnarsdóttir A Sandblom G Arnbjörnsson E Larsson LT Quality of life in adults operated on for Hirschsprung disease in childhood. J Pediatr Gastroenterol Nutr 2010;51:160–6. 20453676 10.1097/MPG.0b013e3181cac1b6

[12] Harden RN Bruehl S Perez RS Birklein F Marinus J Maihofner C Lubenow T Buvanendran A Mackey S Graciosa J Mogilevski M Ramsden C Chont M Vatine JJ Validation of proposed diagnostic criteria (the “budapest criteria”) for complex regional pain syndrome. PAIN 2010;150:268–74. 20493633 10.1016/j.pain.2010.04.030 PMC2914601

[13] Kachko L Efrat R Ben Ami S Mukamel M Katz J Complex regional pain syndromes in children and adolescents. Pediatr Int 2008;50()523–7. 19143976 10.1111/j.1442-200X.2008.02625.x

[14] Kao KT Stargatt R Zacharin M Adult quality of life and psychosocial outcomes of childhood onset hypopituitarism. Horm Res Paediatr 2015;84:94–101. 26045297 10.1159/000430863

[15] Lefante JJ Jr Harmon GN Ashy KM Barnard D Webber LS Use of the SF-8 to assess health-related quality of life for a chronically ill, low-income population participating in the Central Louisiana Medication Access Program (CMAP). Qual Life Res 2005;14:665–73. 16022060 10.1007/s11136-004-0784-0

[16] Maruish ME, User's manual for the SF-36v2 health survey 3rd ed Lincoln: QualityMetric Incorporated, 2011 p. 6–8. Available at: https://cdn-aem.optum.com/content/dam/optum/resources/Manual%20Excerpts/SF-36v2_Manual_Chapter_1.pdf. Accessed September 25, 2019.

[17] Shah RD Suresh S Chronic pain management in children and adolescents. In: Benzon HT Raja SN Fishman SM Liu SS Cohen SP, eds. Essentials of pain medicine. 3rd ed Philadelphia: Elsevier, 2018 p. 325–6.

[18] Sherry DD Wallace CA Kelley C Kidder M Sapp L Short- and Long-term outcomes of children with complex regional pain syndrome type 1 treated with exercise therapy. Clin J Pain 1999;15:218–33. 10524475 10.1097/00002508-199909000-00009

[19] Stein MB Barrett-Connor E Quality of life in older adults receiving medications for anxiety, depression, or insomnia. Am J Geriatr Psychiatry 2002;10:568–74. 12213691

[20] Tan EC van de Sandt-Renkema N Krabbe PFM Aronson DC Severijnen RSVM Quality of life in adults with childhood-onset of complex regional pain syndrome type 1. Injury 2009;40:901–4. 19524904 10.1016/j.injury.2009.01.134

[21] Turner-Bowker DM Bayliss MS Ware JE Jr Kosinski M Usefulness of the SF-8 Health Survey for comparing the impact of migraine and other conditions. Qual Life Res 2003;12:1003–12. 14651418 10.1023/a:1026179517081

[22] User's manual for the SF-36v2 health survey, figure 2.1. Available at: https://cdn-aem.optum.com/content/dam/optum/resources/Manual%20Excerpts/SF-36v2-Health-Survey-Measurement-Model.pdf. Accessed October 15, 2019.

[23] Ware JE Kosinski M Dewey JE Gandek B How to score and interpret single-item health status measures: A manual for users of the SF-8 health survey. Lincoln: QualityMetric Incorporated, 2001.

[24] Wilder RT Management of pediatric patients with complex regional pain syndrome. Clin J Pain 2006;22:443–8. 16772799 10.1097/01.ajp.0000194283.59132.fb

