# Supplementary figures and images for: Outcome in young adults who were diagnosed with complex regional pain syndrome in childhood and adolescence
Source: Pain Rep. 2020 Oct 12;5(6):e860. doi: 10.1097/PR9.0000000000000860 (PMC7553401; doi:10.1097/PR9.0000000000000860)

## Supplemental Figure 2a

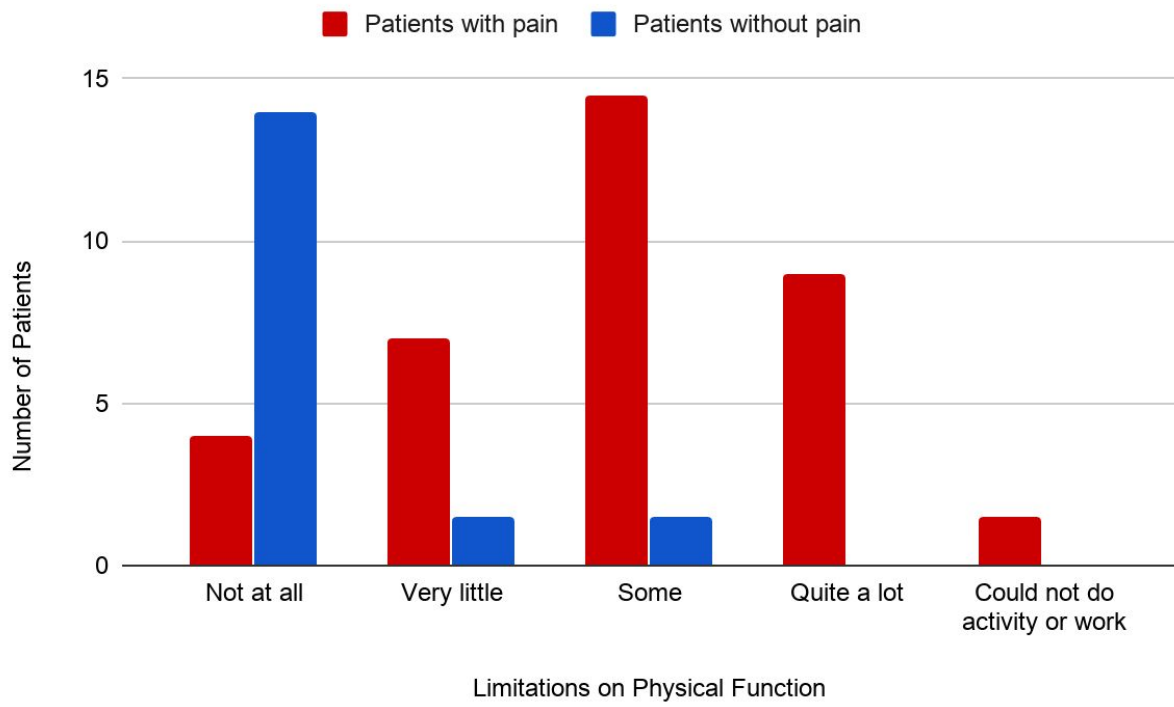

Supplement: SUPPLEMENTARY MATERIAL [file painreports-5-e860-s001.pdf]
